# The Feasibility and User Experience of a Program of Progressive Cued Activity to Promote Functional Upper Limb Activity in the Inpatient Rehabilitation Setting with Follow-Up at Home

**DOI:** 10.3390/app15063010

**Published:** 2025-03-11

**Authors:** Kimberly Bassindale, Sarah Golus, Jake Horder, Maureen Winkoski, Meghann Sytsma, Whitney A. Morelli, Maura Casadio, John McGuire, Robert A. Scheidt

**Affiliations:** 1 Department of Physical Therapy and Human Movement Sciences, Northwestern University, Chicago, IL 60611, USA; 2 Department of Biomedical Engineering, Marquette University, Milwaukee, WI 53201, USA; 3 Department of Physical Medicine & Rehabilitation, Medical College of Wisconsin, Milwaukee, WI 53226, USA; 4 Department of Biomedical Engineering, Università di Genova, 16126 Genova, Italy

**Keywords:** stroke, inpatient rehabilitation, wearable devices, patient-centered therapy, usability, motivation, satisfaction, user experience, mobile health

## Abstract

Although upper limb impairment is one of the most common deficits post-stroke and contributes substantially to diminished functional independence, many survivors receive low dosages of upper limb task training in the inpatient setting. This study evaluates the feasibility and user experience of a progressive-challenge cued activity program, delivered via wearable technology, to promote upper limb activity in an inpatient rehabilitation facility (IRF) post-stroke. Participants (*N* = 30) wore our wearable system *Souvenir*, which provided vibrotactile cues to prompt activity in the more-involved arm during idle time. Compliance with the program was high (94% in the IRF), and the system successfully prompted increased activity, as evidenced by significantly higher post-cue response rates compared to pre-cue activity rates (mean difference = 35.1%, *t*(28) = 9.398, *p* < 0.001). User experience was positive, with participants reporting high usability, satisfaction, and motivation. Follow-up data collected in unstructured home settings (*n* = 23) demonstrated continued high compliance (96%) and favorable user experience. These findings suggest that *Souvenir* and its cued activity program can effectively convert idle time into therapeutic activity while minimizing caregiver burden. Future research should focus on enhancing user engagement and evaluating the clinical efficacy of this approach in improving functional outcomes post-stroke.

## 1. Introduction

Stroke is the leading cause of disability in the United States, with an estimated 9.4 million stroke survivors alive today [1]. Although stroke survival rates have improved in recent decades [2], the result has been a growing population of survivors living with debilitating mobility deficits. Among these, upper limb impairment is one of the most common deficits, affecting as many as 80% of stroke survivors [3,4]. The loss of upper limb function contributes substantially to an inability to perform activities of daily living (ADLs), lending to diminished functional independence and a higher burden of care. To regain functional mobility, intensive therapy interventions are most effective if delivered in the early weeks and months following stroke, when the potential for neuroplasticity is greatest [5]. Subacute stroke survivors expected to regain significant function are often referred to inpatient rehabilitation facilities (IRFs) [6], where they receive high-frequency, intensive rehabilitation services—typically at least 3 h of therapy per day, 5 days a week.

Despite the profound impact of upper limb impairments on functional independence, interventions to restore upper limb function are not the primary focus of stroke rehabilitation in IRFs. Observational studies of stroke rehabilitation indicate that IRFs prioritize lower limb treatment, particularly gait training, while devoting far less time to upper limb interventions [7]. While improving lower limb function is undeniably important in regaining mobility, this emphasis may overlook the vital role upper limb function plays in functional independence and quality of life poststroke [8,9]. Gait training is often prioritized in IRFs to meet mobility-related goals for discharge, but this focus should not detract from the potential stroke survivors have for upper limb recovery during this favorable window of time. The repeated practice of upper limb movements directly related to daily activities—an intervention known as high-repetition task-specific training—is highly effective in improving upper limb motor function poststroke [6,10–12]. However, providing sufficient high-repetition task training dosage in IRFs remains a challenge; previous studies have shown that patients in IRFs engage in an average of only 8 to 44 min per day of task specific upper limb training [13], with some accounts of as few as 4 min per session [14]. Although no guidelines exist for an optimal dosage of high-repetition task-training post-stroke, whether patients are truly receiving “high repetition” is also disputable, with some studies estimating as few as 23–32 repetitions on average in a session [14]. In any event, the average dosage of upper limb training that patients receive in IRFs is unlikely to be sufficient to achieve meaningful improvements in upper limb function [14].

Another key motivation for addressing upper limb impairments early in recovery is the prevention of learned non-use, wherein stroke survivors avoid using their more involved (MI) upper limb and enter a deleterious cycle of worsening function [15,16]. This phenomenon, which is distinct from impaired motor function, is particularly concerning for patients who retain residual motor capacity of the MI upper limb after a stroke but risk losing it due to learned non-use. Several factors may contribute to learned non-use, such as hemi-spatial neglect, a sense of urgency to complete a task using the less involved (LI) limb instead, or low motivation to use the MI limb (e.g., if the non-dominant side is affected by stroke). The current state-of-the-art approach to preventing learned non-use is constraint induced movement therapy (CIMT), which is an interventional program that uses elements of high-repetition task-training of the MI upper limb while also constraining the LI side. The program involves 6 h of training per day across 2 weeks under the guidance of a trained clinician [17]. CIMT has shown clinically meaningful improvements in MI upper limb function that can persist long after intervention [18]. However, widespread adoption of CIMT in clinical practice has been limited by the intensive time demands it places on clinicians [19]. Given the increasing pressure on healthcare resources and clinician time [20,21], increasing total therapy time in IRFs to include more supervised upper limb intervention is highly improbable. Likewise, allotting a greater percentage of existing therapy time on upper limb recovery would come at the expense of other important interventions. There is a pressing need for interventions that engage the MI upper limb early in recovery while also minimizing clinician oversight.

A viable alternate solution would be to leverage each patient’s downtime between therapy sessions to participate in upper limb task practice. Sedentary behavior among stroke survivors is a known concern in IRFs; stroke survivors have been reported to spend up to 74% on average participating in sedentary activities like lying in bed or sitting in a chair watching television [22]. Capitalizing on this underutilized downtime would serve to both limit sedentary behavior and to engage the patient in meaningful upper limb task practice. Such interventions could reasonably be implemented without disrupting the established clinician workflow of scheduled therapy sessions if the intervention requires little to no clinician oversight.

Wearable technology is increasingly recognized as a potentially effective means by which to deliver interventions in environments with limited clinical resources. The implementation of rehabilitative wearable technology into daily life is a realistic prospect, especially given the widespread use of motion sensor technology in commonly owned devices like cellphones and fitness watches. In quite simple applications, wearable motion sensor technology can provide information on the quantity and quality of upper limb movement post stroke [23,24]. Recent use cases for wearable technology have explored the ability to encourage the functional use of the MI upper limb in chronic home settings [25–31] and subacute IRF settings [32–35]. Much of the prior work aimed at promoting increased upper limb movement poststroke has relied on prompts or cues to encourage patients to engage their affected limbs. For example, Da-Silva et al. (2018) prompted stroke survivors to perform pre-selected impaired upper limb tasks with vibration cues delivered to a motion sensor system placed at the wrist [32]. Wearers were prompted once every 1–4 h to encourage the practice of purposeful impaired arm movements, resulting in a total of 3–12 cues per day. Wei et al. (2019) [33] tested the efficacy of prompting activities with 5 repetitions cued every 10 min for 3 consecutive hours, resulting in a total of 90 cues per day. These studies highlight the variability of cue dosage delivery using wearables in IRFs, with intervals ranging from several hours to minutes between each cue. To our knowledge, no study has investigated the effects of delivering larger dosages of cues at higher frequencies (i.e., more than 100 repetitions per day with less than a minute between cues), a strategy more closely aligned with the principles of high-repetition task training.

In this study, we evaluate the feasibility and user experience of a graded-challenge exercise program and associated wearable technology that delivers a high dosage of activity cues and monitors upper limb movement in stroke survivors in an IRF setting. Previous pilot testing conducted with a small cohort of chronic stroke survivors showed positive reception of the system in home settings [27]; here, we evaluated the system in the early days and weeks after stroke. The wearable system aims to promote high-repetition, graded-challenge activities (60 cues per 30 min session, three times per day) that can be tailored to the needs of individual survivors of hemiparetic stroke. The clinician-developed exercise program can be used by patients with a broad spectrum of upper limb impairments, including those who are moderately to severely impaired. This is important because many current interventions to address upper limb function poststroke, such as CIMT, cannot be performed with these individuals. This study captured user experience using validated quantitative surveys that measure motivations to engage with the system, perceptions of system usability, and experiences with the technology. Quantitative movement data were collected using motion trackers worn on both wrists to estimate compliance with the cue responses.

## 2. Materials and Methods

### 2.1. Participant Recruitment

This study was conducted at the inpatient rehabilitation facility (IRF), Froedtert Bluemound Rehabilitation Hospital, Milwaukee, Wisconsin, USA, from 2022 to 2024. All procedures received institutional approval from Medical College of Wisconsin (PRO-42316) and Marquette University (Reliance 21-082, HR-4044) in accordance with the Declaration of Helsinki. This study was conducted in accordance with the Declaration of Helsinki and approved by the Institutional Review Board of Medical College of Wisconsin (PRO-42316, approved 16 December 2021) and Marquette University (Reliance 21-082, HR-4044, approved 3 December 2021) for studies involving humans.

**Figure 1.**
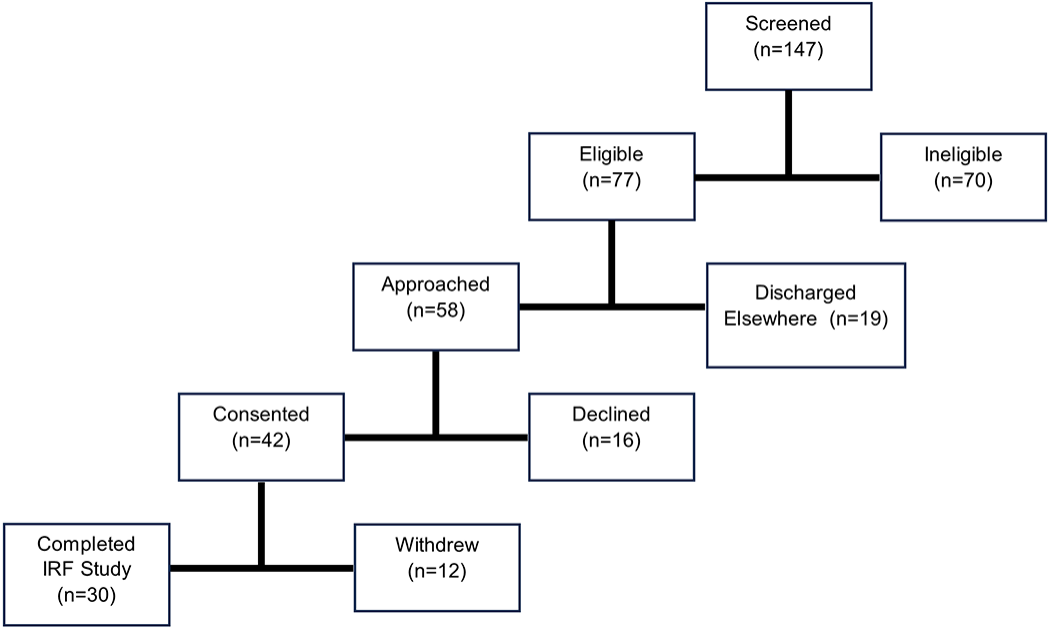
The study recruitment process illustrated, beginning with initial screening of potential participants and followed by eligibility assessment. Of the 77 eligible participants, 58 were approached, and 42 consented to participate in the study. A total of 30 participants completed the inpatient phase of the study.

Medical charts from 149 admitted stroke survivors were screened for study eligibility by a member of the research team (Figure 1). Inclusion criteria included unilateral stroke within the last 30 days, age ≥ 18 years old, and English language speaking. Exclusion criteria included inability to give informed consent or follow two-stage instructions, severe cognitive impairment indicated by a score ≤9 on the Montreal Cognitive Assessment (MoCA), or concurrent illness or injury limiting the capacity to conform to study requirements. Of the participants screened using electronic health records, 77 were eligible for the study. Eligibility and intent to approach for consent was then acknowledged with the patient’s attending physician. Of those eligible, 58 participants were approached by a study team member to describe the study along with its potential risks, whereas the remaining 19 were discharged home or to an outside facility. For those who expressed interest, a study team member administered the MoCA to confirm cognitive eligibility prior to conducting informed consent. The 42 participants who expressed interest all satisfied the cognitive screening criteria and provided written, informed consent to participate in this study. All participants were recruited within an 8-month period for an overall recruitment rate (number of participants consented/months recruiting) of 5.25 participants/month.

After consenting, a total of 30 participants (12 female) completed the study protocol, and 12 participants withdrew (attrition rate: 28.6%). Reasons for withdrawal included disliking the vibrotactile cues after initial demonstration with the device (4), disliking the cues after 1–2 days of use (5), transfer of care to outside facility (1), complex therapy schedule (1), and physician withdrawal (1). Midway through the study, our team recognized the large withdrawal rate due to participants disliking the sensation of the vibrotactile cues. We therefore implemented a protocol amendment that allowed us to provide potential participants with a demonstration of the devices prior to consenting. Once implemented, no participants withdrew from the study due to disliking the cues.

### 2.2. Materials

**Figure 2.**
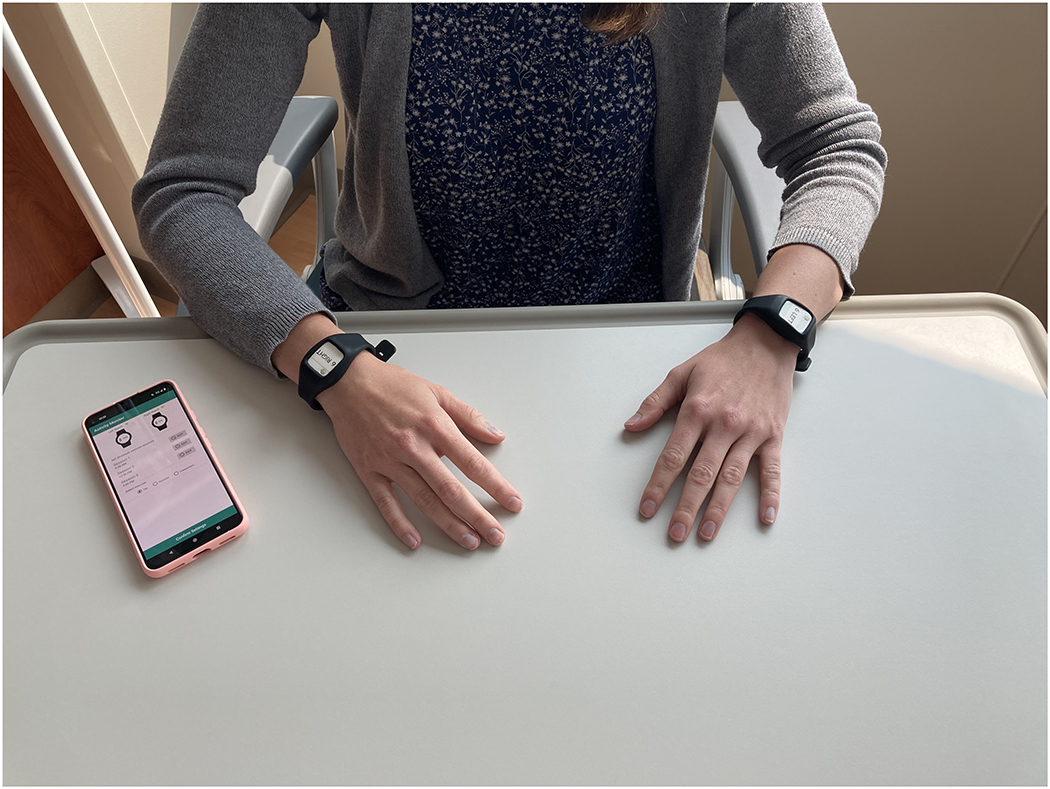
*Souvenir* comprises: two MetamotionR+ wearable activity monitors housed in silicone bands and worn on each wrist, an inexpensive Android smartphone with Bluetooth 5.0 capability, and a custom smartphone app that manages activity scheduling and data storage.

The activity monitoring system used in this study comprises two low-cost MetaMotionR+ wearable activity monitors (MBIENTLAB; San Francisco, CA, USA) housed in silicone bands [one worn on the more-involved (MI) wrist and one on the less-involved (LI) wrist], an inexpensive Android smartphone with Bluetooth 5.0 capability (Google Pixel 5A), and a custom smartphone app that manages activity scheduling and data storage (Figure 2). The wrist-worn MetaMotionR+ devices each include a 3-axis accelerometer, 3-axis gyroscope, and 3-axis magnetometer. The MetaMotionR+ devices also include a vibrating coin motor that provides vibrotactile cues to participants on both wrists when it is time to perform activities. For this study of system feasibility, we sampled the 3-axis accelerometers (16-bit resolution: 1.2 × 10^−4^ g; non-linearity: 0.5% full scale range) at a modest rate of 12.5 samples per second to minimize energy consumption and to allow for rapid upload of files containing the 8 h of time-stamped data. We call the activity monitoring system *Souvenir* because it is designed to remind its users to perform therapeutic activities.

### 2.3. Experimental Protocol

The study was performed in three phases: baseline assessment, IRF feasibility testing, and follow-up at-home activity monitoring.

#### 2.3.1. Baseline Assessment Phase

The following assessments were conducted by a trained member of the study staff: the Fugl–Meyer Assessment of Upper Extremity Sensorimotor Impairment (FM-UE [36]) sections A through D, which yield a total motor function score up to 66 possible points; the FM-UE Section H, which yields a total sensation score ranging up to 12 possible points; and right and left-handed grip strength using a JAMAR hand grip dynamometer [37].

After clinical testing, the study staff demonstrated the system and its nominal use. During this demonstration, we verified that all participants were able to feel the cues on at least one of the two arms.

#### 2.3.2. IRF Feasibility Testing Phase: Progressive-Challenge Cue Activity Program and Passive Activity Monitoring

Study personnel met briefly (i.e., typically < 5 min) with each participant twice per day in the IRF. Each morning, participants were instructed to wear both devices on their wrists and to keep the paired cell phone within proximity (about 25 ft) for a total of 8 h. Within this 8 h activity monitoring period, three 30 min cued activity sessions were scheduled. In these sessions, the system continued to monitor arm activity while the MetaMotionR+ devices delivered haptic vibration cues every 30 s. Thus, each participant was to respond to a total of 180 cues each day.

**Figure 3.**
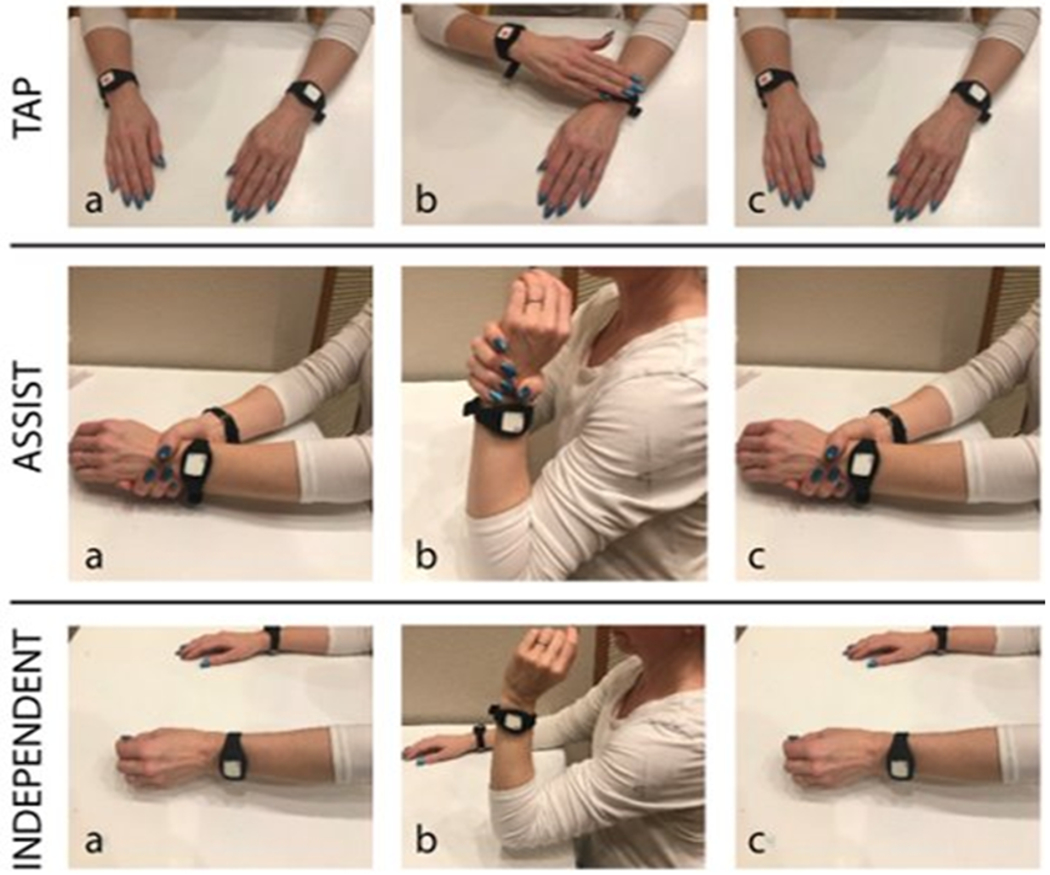
Progressive challenge exercise program ranging from easiest (tapping; top) to more challenging (independent; bottom): (1) tap—the user simply draws their attention to the MI side by tapping their MI arm with their LI hand, (2) assist—the user guides their MI elbow through its full flexion and extension range of motion using their LI hand for support, or (3) independent—the user performs independent active elbow flexion and extension on their MI side without assistance from the LI arm and hand [27]. Labels a through c indicate the temporal order of actions for each exercise.

The cues prompted participants to complete one of three activities (Figure 3): (1) tap—the user simply draws their attention to the MI side by tapping their MI arm with their LI hand, (2) assist—the user guides their MI elbow through its full flexion and extension range of motion using their LI hand for support, or (3) independent—the user performs independent active elbow flexion and extension on their MI side without assistance from the LI arm and hand. All participants began the study by performing the Tap activity on Day 1. They progressed in difficulty on subsequent days at their own pace and with study team guidance. Participants completed a minimum of 1 day of an activity stage prior to progression. Written instructions and pictures describing each activity were offered to all participants.

*Souvenir*’s smartphone application requires the user to input the start times for three 30 min cued activity sessions during times when the participant is expected to be idle (i.e., between therapy sessions and/or other appointments). Study staff reviewed therapy schedules with the participant and collaborated to determine suitable cued activity session times. Study staff encouraged participants to independently determine session times to maximize autonomy with the system.

Participants were allowed to doff the devices prior to showering or other event of water submersion but were asked to don the devices again once their skin was completely dry. In addition, participants were encouraged to doff the devices if they caused excessive skin irritation or discomfort, if they interfered with rescheduled therapy sessions, or if they became an annoyance. Nursing staff were informed of their patient’s participation in the research study and were provided with education on the study protocol to assist participants with donning and doffing the devices if needed.

After each daily 8 h period, study personnel met briefly with the participant to assist as needed to doff the devices, check the participant’s skin where the devices were worn, and document the participant’s subjective comments about the devices. Study staff then removed the devices from the participant’s room and charged them for the next day’s use.

#### 2.3.3. Final Day Prior to Discharge from IRF

The participant completed three surveys to assess user experience with the devices on the last day of wearing the devices in the IRF setting (typically one day prior to discharge). These included (1) the System Usability Scale (SUS; [38]), which assesses the usability of the activity cueing system and monitoring system within the context of encouraging therapeutic arm activity; (2) the Quebec User Experience and Satisfaction with Technology (QUEST; [39,40]), which evaluates user satisfaction with the system and its delivery by the research team; and (3) the Intrinsic Motivation Inventory (IMI; [41]), which assesses the subjective experience of motivation in response to the vibrotactile cues delivered by the system. Questions from the IMI span six psychosocial dimensions, including interest/enjoyment, effort/importance, value/usefulness, perceived choice, perceived competence, and pressure/tension. Survey length and scores are summarized in Table 1. User experience surveys were distributed either physically on paper or verbally in person in the IRF. Participants received a modest stipend for completing the surveys after all study materials were retrieved.

#### 2.3.4. Follow-Up Phase at Home: Attrition and Passive Activity Monitoring

Participants were contacted between 1 and 6 months post-discharge from the IRF with an offer to participate in a follow-up study at home. We performed this follow-up study to assess the feasibility of re-engaging with participants following discharge from IRF and to assess compliance with device wear in unstructured home settings. Both assessments could inform future efficacy studies requiring a longitudinal protocol spanning recovery both in the IRF and at home. Follow-up sessions were scheduled either at the participant’s home or at Froedtert Hospital. Sessions conducted at Froedtert Hospital were completed before or after a participant’s routine medical appointment out of convenience to the participant. During the visit, baseline tests were reassessed, and then participants were given an instructional packet with written instructions and step-by-step pictures describing how to complete at-home silent monitoring. Specifically, participants were instructed to wear the devices for 8 h on 2 separate days using a phone application that monitored activity motion only; no vibrotactile cues were delivered during this phase of the study. Pre-paid shipping boxes were provided to the participants to allow them to mail the devices back to study personnel after use. Lastly, after the two days of device use, participants again completed the SUS and QUEST surveys to assess user experience with the silent monitoring application. Surveys were completed verbally over the phone or electronically via a web browser using REDCap (Research Electronic Data Capture; [42,43]) tools hosted at the Medical College of Wisconsin.

#### 2.3.5. Data Curation and Analysis

Each MetaMotionR+ device streams to the smartphone time-stamped, accelerometry values for the X, Y, and Z axes in gravitational units (1 g = 9.8 m/s^2^); these data are accumulated into a data file throughout each day of data collection. During IRF use, the system also creates a file with time-stamped events indicating when vibrotactile cues are delivered by the MetaMotionR+ devices; no cues are provided during the at-home phase of the study. Data files are stored locally on the smartphone and are also uploaded automatically (without user intervention) to a HIPAA-compliant online storage site at the end of each day of study participation.

We used the time-stamped accelerometry data from the two wrist-worn MetaMotionR+ devices to compute two primary outcome variables pertaining to patient compliance with study procedures; these two factors are important determinants of the practical feasibility of using the *Souvenir* system to encourage therapeutic activity after stroke.

We defined system wear time as the overall amount of time each day that the patient wore the two MetaMotionR+ devices while maintaining proximity to the smartphone such that the Bluetooth wireless connections were maintained. Specifically, to compute system wear time, we analyzed the two MetaMotionR+ accelerometer data files for gaps between successive data points greater than or equal to 60 s duration. We reported system wear time as the percentage of the intended 8 h daily wear time without a data gap in either wrist device. We regarded system wear time as a measure of compliance to protocol, with compliance reported as the summation of all instances with less than 60 s of consecutive missing data divided by the total monitoring time (multiplied by 100 to yield a percentage).

We computed the activity cue response rate by determining the number of cue indicators eliciting a significant activity response. As described previously [27], several post-processing steps were needed to compute the activity cue response rate. We used the data timestamps to synchronize and resample the two sets of MetaMotionR+ accelerometer data and the system cue event timestamps to yield a synchronous and simultaneously sampled bilateral motion dataset. The resulting accelerometry values along each axis were filtered using a bandpass window of 0.25 Hz to 2.5 Hz (MATLAB function bandpass); this window was chosen to be similar to windows used by Bailey et al. 2014 to filter out the static effect of gravity as well as other sources of extraneous motion or noise [44]. Using these filtered data, we calculated the magnitude of acceleration measured at each wrist using the Euclidean norm. Acceleration magnitudes were then summed within each 1 s epoch to yield a time series of each arm’s acceleration magnitude for every second of monitoring throughout the day. We then transformed the acceleration magnitude values into an intermediate variable called activity counts using a thresholding approach [27] similar to those described by Bailey et al. (2014) [44] and Uswatte et al. (2013) [45,46]. We computed activity cue response rate by counting the number of vibrotactile cues immediately followed (within 5 s) by a period of arm activity greater than or equal to 1 s and then dividing that sum by the actual number of cues provided. Data processing of activity monitoring data and compliance calculations was completed using the MATLAB computing environment (version R2021a; the MathWorks, Inc., Natick, MA, USA).

Finally, we computed three primary outcome variables pertaining to subjective user experiences using the SUS, IMI, and QUEST surveys. Survey scores were computed using each survey’s standard scoring method. Specifically for the SUS, negatively worded items were subtracted from the maximum scale value of 5 before summing all items. The sum of all ten item scores was multiplied by 2.5 to give an overall score ranging from 0 to 100; a value of 68 is considered the cut-off threshold for passible usability [38]. Our 37-item version of the IMI was constructed using language specific to determining motivation related to performing therapeutic activities in response to haptic cues. Responses within each of the six subscales were averaged for each participant to produce a score ranging from 1 to 7 for each of the six dimensions of the IMI. The QUEST score is calculated simply by taking the average score across all responses. Because there are no established cut-off scores for passability on the QUEST or IMI surveys, the middle value of each scale (3 and 4, respectively) was used.

#### 2.3.6. Statistical Hypothesis Testing

We tested three hypotheses. We first hypothesized that participants would comply with wearing the *Souvenir* system in the IRF; we tested this hypothesis using separate planned one-sided *t*-test to compare system wear times in each phase of the study (IRF; at home) to a nominal minimum threshold of 90% of intended wear time (Hyp 1). We also hypothesized that participants would comply with performing the cued activities by using a planned one-sided *t*-test to compare activity cue response rates (IRF phase only) to a nominal minimum threshold of 90% delivered cues (Hyp 2). Finally, we hypothesized that participants would have positive subjective experiences using the system while in both IRF and home settings (Hyp 3). To test this last hypothesis, we used separate one-tailed *t*-tests to compare cohort survey responses to the survey-specific cut-off scores described above.

We also performed secondary analyses of cue compliance as a function of time during the day and as a function of cued activity type; we report the results of these exploratory analyses for use as baseline statistics to inform the design of future studies intending to improve compliance with, and/or efficacy of, cued activity therapeutic interventions after stroke. Statistical analysis was performed using RStudio version 4.1.1 with statistical significance set at *p* < 0.05 for the planned analyses and a family-wise error rate alpha = 0.05 for secondary analysis using Bonferroni correction.

## 3. Results

### 3.1. Feasibility and User Experience in an Inpatient Rehabilitation Facility

#### 3.1.1. Compliance

Participants wore the devices on average 5.6 ± 2.6 (mean ± standard deviation) days in the IRF. Of the resulting 167 total days of participant engagement, data from 18% of the days were unusable or otherwise incomplete. Incomplete data collection occurred due to either environmental constraints or technical errors. Environmental constraints included (1) changes in therapy schedule and (2) acute discharge from the IRF due to a change in the participant’s medical status. Technical errors included (1) an undesirable software interaction from *Souvenir*’s demonstration app onto its cueing and monitoring app causing system event timing interference and (2) system interruptions resulting from Android smartphone system software updates. There were no failures due to battery loss or other causes. The remaining 137 participant days were included for further analysis.

Instances wherein the MetamotionR+ devices dropped their Bluetooth connections with the smartphone were relatively rare. This did happen occasionally, for example, if the patient forgot to bring the cellphone when leaving their room for therapy. We computed compliance time for each device on each day and averaged these values across days for each participant. Average compliance time across participants was 94.0 ± 8.7% and 94.0 ± 8.6% for the MI and LI sides, respectively.

#### 3.1.2. Cue Response Rate

**Figure 4.**
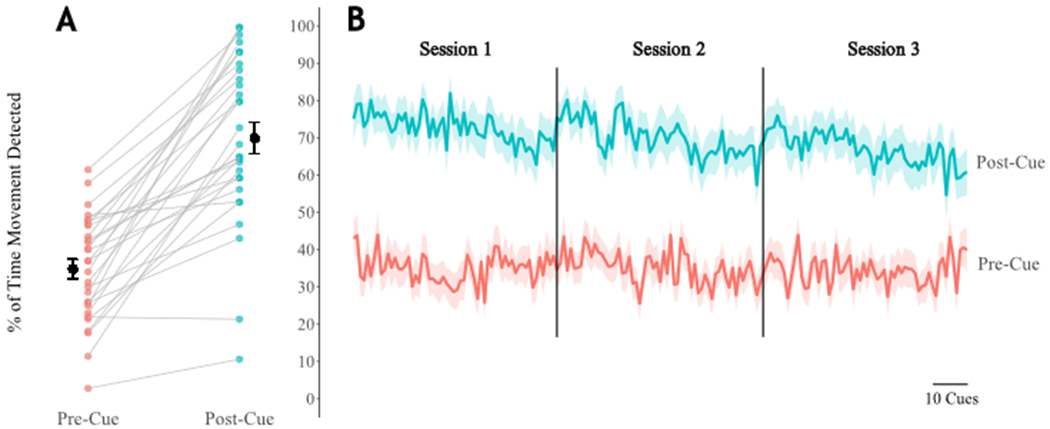
(**A**) Paired average response rates across days for each participant. Pre-cue includes the 5 s of activity prior to cue delivery, and post-cue includes the 5 s following cue delivery. Black dots and lines indicate the overall mean and SEM. (**B**) Average response rates across all days within all participants for cues 1 through 180. See Supplementary Materials for supporting data tables (S4).

Across participants, post-cue response rates averaged 65.95 ± 22.45% (Figure 4A). In contrast, the number of cues with activity present in the 5 s time window preceding the cue (a value we call the pre-cue activity rate) averaged only 34.81 ± 14.12%. To assess the utility of the system in prompting arm activity, we performed a paired *t*-test to compare the activity/response rates between pre- and post-cue intervals and found that post-cue response rates were significantly greater than pre-cue activity rates (mean of the differences = 35.1%, 95% CI = [27.5, 41.8], *t*(28) = 9.398, *p* < 0.001).

#### 3.1.3. User Experience Surveys

**Figure 5.**
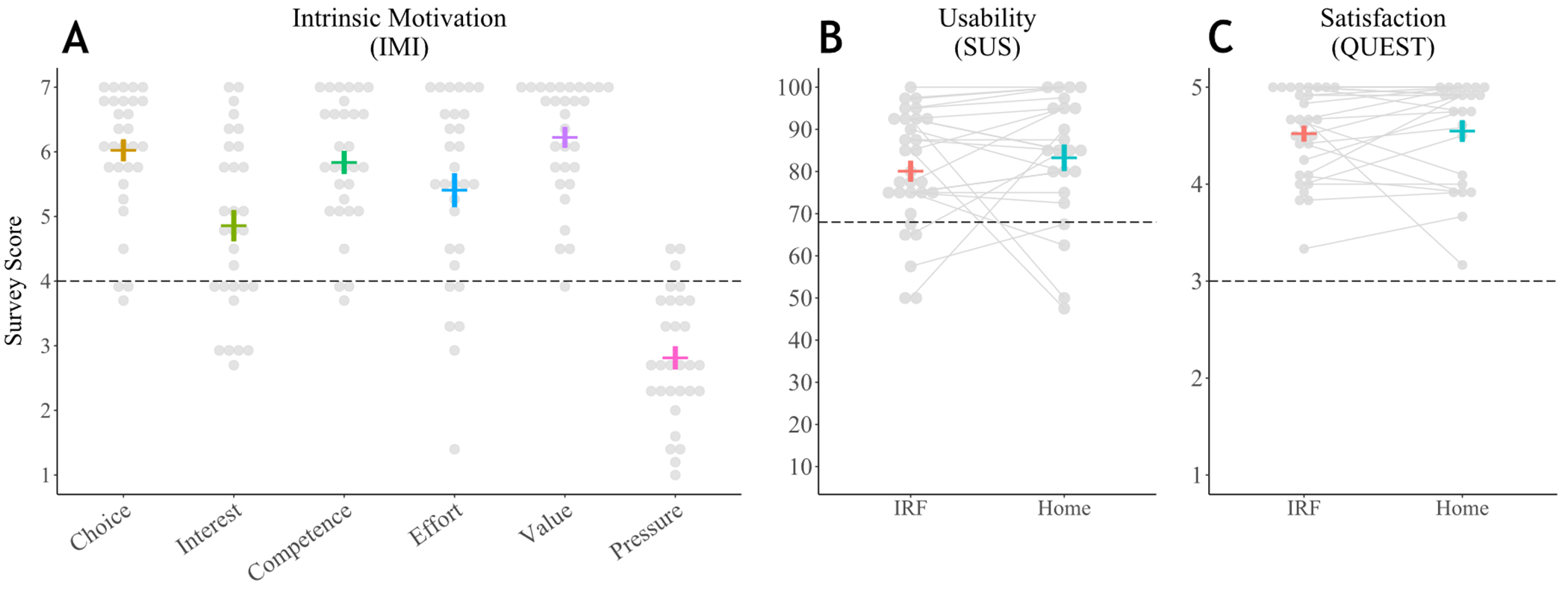
Survey responses provided after system use; dashed lines indicate thresholds for passable scores. Mean scores and SEM are represented by colored horizontal and vertical bars, respectively. (**A**) Intrinsic Motivation Inventory (IMI) completed in the IRF. All scores met passable criteria on average, with value/importance rated highest on average. (**B**) System Usability Scale (SUS) completed in IRF and home setting is labeled going from left to right. Dashed lines in figures (**A**–**C**) indicate passable cut-off scores for the IMI, SUS, and QUEST surveys, respectively. Mean scores are represented by horizontal bars, and SEM is represented by vertical bars. See Supplementary Materials for supporting data tables (S4).

**Figure 6.**
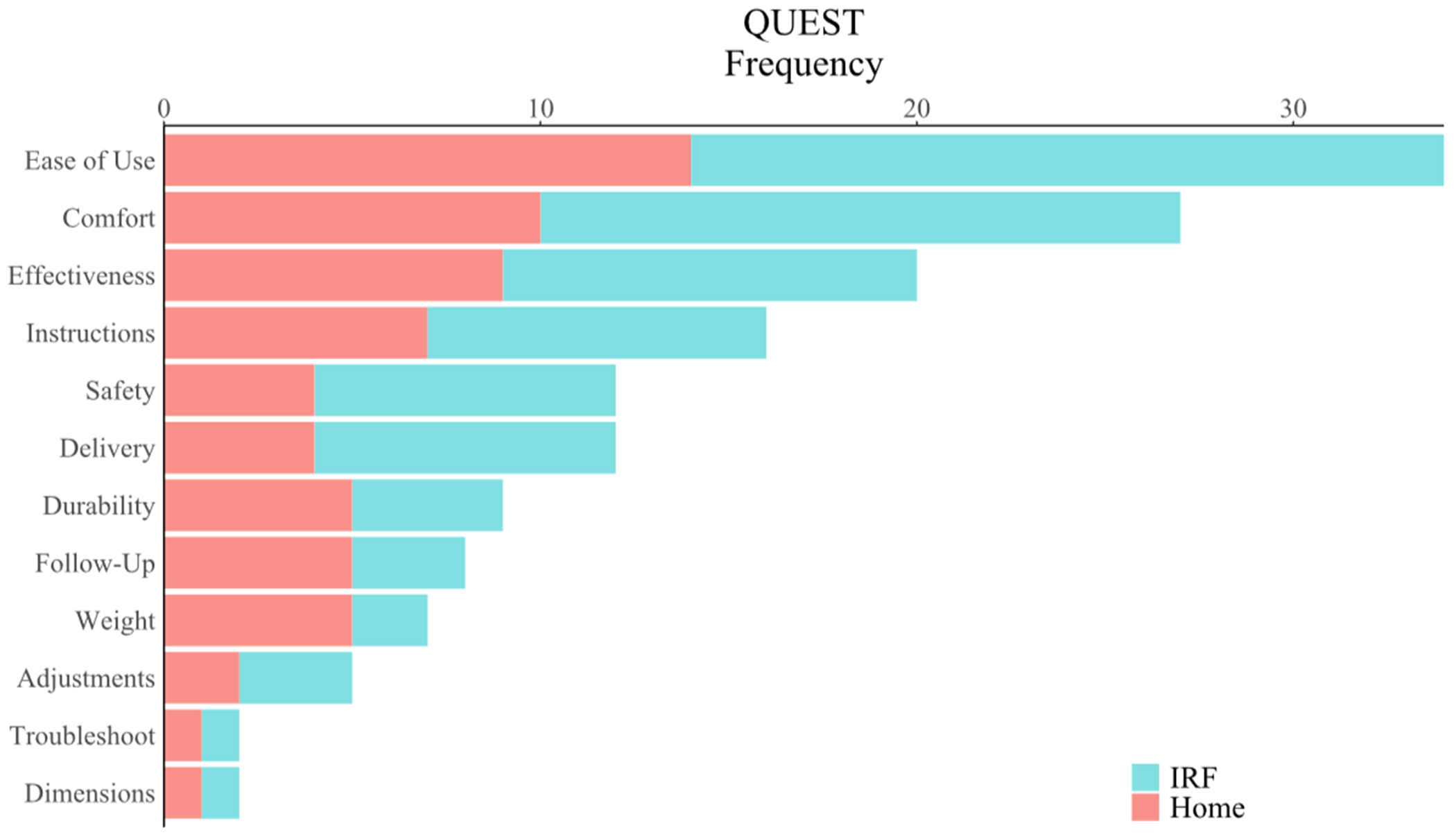
Categories of the Quebec User Experience and Satisfaction with Assistive Technology (QUEST) users identified as most important for promoting a positive user experience. Ease of use, comfort, and effectiveness were identified most frequently out of the categories in both the IRF and home settings. See Supplementary Materials for supporting data tables (S4).

We next assessed the system’s overall user experience during IRF use by analyzing participant responses to surveys of the system’s usability, satisfaction, and intrinsic motivation. We performed a one-tailed, one-sample *t*-test to compare the mean participant SUS scores to the accepted threshold of 68 for system usability. Mean participant SUS scores (80.1 ± 13.8) were significantly higher than the acceptable threshold (90% CI = [75.8, 84.3], *t*(29) = 4.80, *p* < 0.001), demonstrating acceptable usability of the system in the IRF (Figure 5B). Similarly, we used one-tailed, one-sample *t*-test to compare the mean QUEST score to the midpoint threshold value of 3, which separates positive from negative assessments. The mean QUEST score (4.52 ± 0.46) was significantly higher than the threshold (90% CI = [4.38, 4.66], *t*(29) = 6.23, *p* < 0.001), suggesting that the system and its delivery were satisfying among stroke survivors in the IRF (Figure 5C). Of the 12 items from which users were to select the three of most importance to them for determining satisfaction, ease of use, comfort, and effectiveness were selected most often, whereas troubleshooting and dimensions were selected least often in the IRF (Figure 6).

For each of the categories of the IMI, we completed a one-tailed, one-sample *t*-test to compare the mean IMI category score to the midpoint threshold value of 4. The mean IMI scores for value/usefulness (6.2 ± 0.9), perceived choice (6.0 ± 0.9), perceived competence (5.8 ± 1.0), effort/importance (5.4 ± 1.4), and interest/enjoyment (4.9 ± 1.3) all significantly exceeded the threshold value (value/usefulness: 90% CI = [5.95, 6.50], *t*(29) = 13.60, *p* < 0.001, perceived choice: 90% CI = [5.73, 6.32], *t*(29) = 11.75, *p* < 0.001; perceived competence: 90% CI = [5.53, 6.14], *t*(29) = 10.20, *p* < 0.001; effort/importance: 90% CI = [4.96, 5.85], t(29) = 5.34, *p* < 0.001; interest/enjoyment: 90% CI = [4.44, 5.27], *t*(29) = 3.51, *p* < 0.001) (Figure 5A). The mean score for the category of pressure/tension (2.8 ± 1.0) was significantly lower than the threshold value of 4 (90% CI = [2.51, 3.12], *t*(29) = −6.57, *p* < 0.001), which reflects a positive user experience in this singular category.

### 3.2. Feasibility and User Experience During Follow-Up at Home

#### 3.2.1. Compliance

Of the 30 participants who completed IRF phase of the study, 23 (76.7%) completed home follow-up. The remaining seven did not participate due to the inability of research staff to contact the participant after multiple attempts (four), a change in medical status (two), and moving out of state (one). Follow-up visits were completed an average of 124.2 ± 33.1 days following discharge from IRF. Participants were given the option to receive study equipment at their home (*n* = 6) or at Froedtert Hospital before or after a clinical visit (*n* = 17). In the 44 participant days of at-home silent monitoring, 81.9% of potential silent monitoring data were collected. The reasons for missing data were less clear in the home setting compared to the IRF. Only one participant called to report technical issues with the system, which resulted in less than 1 h of monitoring time for both days for that participant. No other technical failures were reported by users, although we suspect such issues were probable based on IRF findings. The average compliance time across participants was 96.3 ± 9.9% and 94.6 ± 12.9% for the MI and LI sides, respectively. All devices were successfully returned via pre-paid packaging by the participants.

#### 3.2.2. User Experience Surveys

We compared the mean participant SUS score at follow-up (83.3 ± 15.2) to the passability threshold value of 68 (90% CI = [77.81,88.71], *t*(22) = 4.81, *p* < 0.001) and found that the system had acceptable usability in home settings (Figure 5B). We similarly compared mean QUEST score (4.5 ± 0.5) to the midpoint threshold value of 3 (90% CI = [4.35,4.74], *t*(22) = 4.87, *p* < 0.001), finding that the system and its delivery were satisfying among stroke survivors in the home settings (Figure 5C). As with the survey results obtained after the IRF phase of this study, participants selected *ease of use, comfort*, and *effectiveness* as items of most importance for determining satisfaction in home settings, whereas *troubleshooting* and *dimensions* were selected least often (Figure 6).

## 4. Discussion

This study characterized the feasibility and user experience of a progressive-challenge cued activity program designed to encourage upper limb activity during idle time in an inpatient rehabilitation facility (IRF) post-stroke. We also performed a follow-up assessment of the feasibility of activity monitoring in home settings. The *Souvenir* system and its cued activity program demonstrated utility in the IRF by delivering cues with a high success rate and by prompting increases in upper limb activity, as indicated by increased limb activity (response rates) within a short time window immediately following cue delivery. The system and its cued activity program provided a positive use experience in the sense that users found them to be usable, motivating, and satisfying to use in the IRF. The same users also found the activity monitoring application usable and satisfying to use during follow-up assessment in home settings. These findings are important because they demonstrate that a program of high-repetition progressive challenge cued activity can motivate survivors of hemiparetic stroke to significantly increase the activity of the more involved arm during idle-time in the IRF and that the same system can be used successfully for monitoring arm activity in unstructured home environments.

### 4.1. Reclaiming Lost Opportunities: Current Approaches to Subacute Rehabilitation

IRFs are typically constrained by Medicare reimbursement limitations to provide intensive multidisciplinary therapy up to 3 h/day, 5 days/week. Even though this may seem like a relatively high therapy dosage, subacute stroke survivors in IRFs spend an average of 74% of their daily time sedentary [22]; this exceeds the 55% estimated average sedentary time of children and adults in the general population [47]. Further concerning is that sedentary bouts are often prolonged among stroke survivors, with bouts lasting greater than 1 h accounting for 44% of daily time on average [22]. In adults, there is accumulating evidence that greater sedentary behavior increases the risk for mortality, cardiovascular disease, and type 2 diabetes [48]. Among stroke survivors specifically, sedentary behavior has been associated with an increased risk of stroke recurrence [49]. Multiple definitions exist for classifying behavior as sedentary, but it is generally agreed that sedentary behavior encompasses activities that do not substantially increase energy expenditure above resting levels, such as lying down, sleeping, or sitting while watching television [50]. Reducing sedentary behavior in this population and setting is challenging, particularly because many stroke survivors face cognitive and physical impairments that limit their capacity to independently engage in activity. Inactivity also contributes to the deleterious cycle of learned non-use in the paretic upper limb, leading to worsening functional outcomes over time. Skilled intervention targeting paretic upper limb function, such as task-specific training, has been shown to significantly improve arm function post-stroke [10]. Yet, the usual care dose for upper limb task practice post-stroke has been found to be highly variable, with reported doses ranging only between 8 and 44 min spent on upper limb tasks per day [13]. This situation necessitates a solution that promotes increased upper extremity activity doses without demanding increased personnel resources in an already overburdened system.

Remote wearable technology affords an opportunity to convert sedentary idle time into upper limb activity without significantly increasing the burden on clinician time. Ours is not the first study to capitalize on this idea; a related study by Signal et al. (2023) investigated whether the timing and frequency of occasional vibrotactile “nudges” affect upper limb movement response in a cohort of 20 stroke survivors receiving care in an IRF. The authors found that stroke survivors were more likely to respond to haptic nudges delivered during periods of higher activity (e.g., around lunch) compared to times of typical inactivity [35]. They also reported that haptic nudges induced an immediate increase in upper limb movement, although the effects were limited to a duration of less than 1 min [35]. In contrast, the current study assessed the immediate responses to frequent cues—i.e., in the 5 s immediately preceding and following each delivered cue—to determine if cues successfully elicited movement in the correct limbs. The activity program and wearable system *Souvenir* described in this study are novel in their delivery of high-dose cues that can evoke upper limb activities graded by user functional capacity. The activity program described in this report was specifically designed to allow stroke survivors with severe motor impairments and/or mild to moderate cognitive deficits to participate in upper limb activity in the absence of caregiver oversight. Although we found that cues did successfully elicit upper limb movement as intended (Figure 4A), we also noticed a gradual reduction in the average cue response rate both within a given session as well as over the course of each day (Figure 4B); the results indicate that participant engagement with *Souvenir*’s cued activity program wanes as the day progresses. Our time-varying response rate results, along with findings from Signal et al. (2023), imply there may be an optimal cue dosage (both timing and frequency) that is able to evoke maximal responses. Determining the optimal dosage is complicated because the likelihood of responding to any given cue could be impacted by an aggregate of psychosocial factors post-stroke, such as increases in pain [51], fatigue [52], or depression symptoms. In traditional therapy, clinicians can immediately adapt interventions based on the patient response to maximize therapeutic efficacy, whereas remotely used wearable technology has not reached this capability in the stroke population. Further research is needed to determine the extent to which such psychosocial factors affect the response to cued interventions. Such knowledge could be used to optimize the delivery of remotely delivered interventions with wearable technology.

In the current study, participants found the progressive challenge activity program and the wearable system to be usable, motivating, and satisfying to use in the IRF, as evidenced by average SUS, IMI, and QUEST scores. All IMI categories met passable thresholds in the IRF, although the interest/enjoyment subscale (4.9 ± 1.3 out of 7) scored the lowest among the positively rated categories, which suggests room for improvement on this measure. One potential contributor to the lower interest/enjoyment score could be the modest challenge of our current activity progression program. This initial program was designed to involve simple activities that could be performed at high repetition, that could progress through a gradual increase in challenge, and that could be accessible by stroke survivors with a range of motor and/or cognitive deficits. Although all participants saw value in the cued activity program, as noted by a high average value/usefulness score on the IMI (6.2 ± 0.9), it will, in the future, be important to consider how lower levels of interest might affect adherence to device use, especially if used over the course of weeks or months. Anecdotally, some participants reported wanting to perform an activity more attuned to their specific impairment, such as wrist movement instead of elbow movement. The individualization of activity prescription during stroke rehabilitation is integral to maximize patient engagement and functional recovery. We believe expanding the activities available in *Souvenir*’s activity program—while maintaining the straightforward progression of MI upper limb engagement—may improve patient perceptions of salience and interest in the program, though this must be demonstrated by further research.

Another potential cause of lower interest in *Souvenir*’s current implementation could be the repetitive nature of the activity program. Even though programs involving high-repetition task training have been found to significantly improve upper limb function post-stroke [12], patient adherence to such programs is known to be low, and, when asked, patients often overestimate the frequency and intensity of their exercise bouts [53]. Several researchers have suggested that the challenge of maintaining engagement in repetitive activity might be mitigated through adoption of gamification strategies [54–57], which can be effective at delivering high-repetition neurorehabilitation interventions among stroke survivors [56,58–60]. Gamification involves concepts of dynamic task difficulty adjustment to enhance user motivation and engagement [61]. The successful integration of gamification concepts into *Souvenir*’s delivery of the progressive-challenge activity program could potentially enhance cue response rates throughout the day while additionally offering important insight into behavioral responses to dynamic cued activity in the stroke population.

### 4.2. Opportunities and Future Directions

We quantified cue response rates by applying a static threshold value to acceleration magnitude data from the two arms. This activity detection technique provides a simple quantifiable measure of upper limb movement but, in isolation, does not allow an assessment of movement quality or accuracy. Adding a more complex activity recognition capability to our current system is plausible, as others have reported high accuracy in assessing goal-directed upper limb movement quality with activity recognition algorithms [62,63]. Movement quality information has the potential to provide valuable feedback to users to further engage them in the activity program, especially if movement quality feedback is provided in real-time. In one exemplar study, Schwerz et al. (2022) explored the effects of providing real-time feedback on hand and wrist movement “counts” to chronic stroke survivors using a wearable wristwatch/ring system compared to a control group of survivors that used the devices without feedback. Their system used a threshold method to quantify active movement similar to that used in the current study. The authors found a significant increase in hand counts among the group receiving real-time feedback and a non-significant increase in hand counts in the control group [28]. Stroke survivors who received real-time feedback with complimentary count goal setting were also found to have worn the device more hours per day on average compared to the control group [28]. Those results align with widely accepted views that feedback can positively influence behavior and performance in rehabilitation [10].

Although real-time feedback or more advanced activity recognition features may be advantageous to user experience, drawbacks of these features on an affordable wearable system must be considered. That is, an assessment of movement quality typically requires higher sampling frequencies compared to activity detection alone [64], and the integration of real-time movement recognition into wearable systems likely will require greater processing power (c.f., [65]). Adding these system functions could decrease device wear-time (and therefore system usability) for any given battery capacity. Thus, advanced features must be carefully balanced with system power requirements, and they must demonstrate significant advantages in eliciting a desired behavioral response before adoption into a practical wearable system.

### 4.3. Limitations

This study has a number of limitations. First, our attrition rate was relatively high at 28.6%. The most reported reason for withdrawal (9/12) was due to disliking the vibrotactile cues. To address this concern, we amended our protocol to allow participants to receive a demonstration of the system prior to enrollment; after implementing this change, we had no further dropouts due to this reason. Although we expect this change in protocol to improve attrition for future studies, it will be important to also consider alternative methods of cue delivery to be more inclusive of individuals who will not tolerate the vibrotactile sensation of our current cues. Alternative methods of cue delivery may also expand the accessibility of our system to individuals with greater sensory impairment, such as severe sensory loss or neglect. However, further research is needed, as only two participants in our study had sensory deficits per FMA-UE sensory scores, limiting our ability to draw statistical conclusions about this group. Of note, no participants withdrew from the study due to disliking the progressive-challenge activity program. Despite our attrition rate being higher than expected, we completed our study with a high recruitment rate of 5.25 consents/month. Furthermore, we were able to follow-up with a majority of participants (76.7%) for the at-home phase of the study. By contrast, a systematic review by McGill et al. (2020) reported that median recruitment rates in stroke rehabilitation studies were much lower, with a median recruitment rate in North America of 1.35 consents/month [66].

Attrition rates are known to be highly variable in stroke rehabilitation studies and are dependent on the study location, length, and setting [66]. A systematic review by McGill et al. (2020) consisting of 512 randomized control trials with 28,804 stroke survivor participants found attrition rates ranging between 0 and 83%, with a median of 6% [66]. While our study’s attrition rate is higher than this reported median, it is comparable to that of another study investigating the feasibility of a wearable system among stroke survivors in an IRF (a reported attrition rate of 29%) [34]. The wearable system in that study was designed to provide feedback on upper limb activity and to prompt activity during inactive periods to assist the patient in reaching a clinician-set goal for upper limb movement. From a total of 17 recruited participants, the authors reported that 3 withdrew from the study due to the psychological burden of using the wearable system in combination with other therapies, 1 withdrew due to an allergic reaction to the device straps, and another withdrew due to early discharge. No participants reported withdrawing due to disliking the vibrotactile system, perhaps indicating that feedback and goal setting may reduce attentional focus on the vibrational stimuli themselves but add to patient perceptions of cognitive burden.

We acknowledge, as another limitation of the study, several sources of bias toward a more favorable user experience. Participants who withdrew from the study prior to completing 3 days of the progressive-challenge activity program did not complete user experience surveys. A reasonable critic could argue that those who withdrew due to disliking the vibrotactile cues would have rated the system less favorably compared to those who tolerated the cue sensation. Bias toward more positive user experiences is also inherent to the study in that individuals who are more likely to consent to participating in an activity program using wearable technology may be more likely to perceive it favorably. Given that a high percentage of patients approached for the study consented to participate (72.4%), individuals in the immediate aftermath of stroke may be particularly motivated to use wearable systems promoting independent therapeutic activity.

A general limitation of wearable technology used remotely is the likely possibility of missing data. Our system exhibited relatively high rates of data capture (IRF: 82%; home: 80.9%). Likewise, patient compliance with wearing the devices was high in both settings (IRF: 94%; home: 94–96%). Taken together, these results demonstrate patient willingness to engage with (and *Souvenir*’s ability to conduct) at least 8 h of activity monitoring per day. Errors in the phone application contributing to missing data were easier to diagnose and document in the IPR setting compared to the home settings due to our performance of end-of-day check-ins in the IRF; upon checking data file integrity at the end of the day, study staff were able to detect and respond quickly when automatic Android software updates interrupted cue delivery and activity monitoring. However, such events highlight the need for ongoing system code maintenance to protect against forced obsolescence caused by uncontrollable events like system updates, cloud service API changes, changes in applicable regulations, etc. Enhancing the smartphone app to include error logging and diagnostics could also help identify technical issues, which would be particularly useful for device use in less supervised home settings. Participants received written instructions for using the device at home, including a section on basic troubleshooting to reduce the risk of data loss. We believe the most effective approach to addressing data loss is through technological improvements, rather than placing the burden on the user by adding to these instructions. Lastly, our calculations of compliance did not include an estimation of non-wear compliance in which the user doffs the wristbands while keeping them within proximity of the phone. While computations for this type of non-wear exist [67], they have not yet been validated in the stroke population. Excluding non-wear time from our compliance calculation is unlikely to significantly affect the overall trends observed between pre-cue activity and post-cue response rates because choosing to doff the devices equally impacts pre- and post-cue activity.

Finally, our sample included a higher percentage of participants with relatively high motor and sensory function (reflected by FMA-UE scores) and normal cognition (reflected by MoCA scores), which may limit the generalizability of our results. Many participants achieved the maximum FMA-UE score of 66 despite presenting with coordination deficits and/or strength deficits (reflected by maximum grip strength). Post hoc analyses found no significant differences in user experience survey scores between those with mild versus moderate–severe motor impairments or between those with normal versus mild–moderate cognitive deficits, highlighting that these factors did not significantly impact outcomes in our study (see Appendix A). Although our post hoc analyses did not reveal any significant differences in user experience based on cognition or motor function, we acknowledge that our study was not originally designed to adequately compare these groups. Future research should include larger sample sizes and a wider range of functional stratification (such as patients with severe motor and/or sensory impairments). Doing so could provide more robust evidence that user experience is comparable across cognitive, sensory, and motor function levels.

## 5. Conclusions

Our study contributes to a growing foundation of prior work evaluating the feasibility of wearable technology to promote rehabilitation after stroke [32–35]. To our knowledge, our study is the first to assess the feasibility of delivering a high dosage of vibrotactile cues to motivate a program of cued therapeutic activity of the paretic upper limb during idle time in the IRF. We showed that compliance with the program of progressive-challenged cued activity was high in the IRF after stroke and that the progressive-challenge activity program and wearable cueing system were well-received by stroke survivors. The next step in this line of research will be to explore ways to improve interest/enjoyment in the cued activity program, because this was the lowest subjective user experience score (even though the score was deemed passable). If successful, *Souvenir* and its program of progressive-challenged cued activity will leverage patient idle time post-stroke into therapeutic activity time with minimal impacts on caregiver burden. Follow-up involving system use in a home environment was feasible for this cohort, with 76.7% of participants completing the follow-up protocol. We plan to use these findings to inform the design of a future efficacy study of a progressive-challenged cued activity program to improve clinical outcomes after stroke.

## Supplementary Material

Survey S3. System Usability Scale Survey

Survey S1. Intrinsic Motivation Inventory Survey

Survey S2. Quebec User Experience and Satisfaction with Assistive Technology Survey

S4. CalculatedSurveys_IPRandHome

S4. ForwardResponses_ByParticipant

S4. BackwardResponses_ByParticipant

S4. ResponseRatesBySubject

The following supporting information can be downloaded at: https://www.mdpi.com/article/10.3390/app15063010/s1, Survey S1: Intrinsic Motivation Inventory; Survey S2: Quebec User Experience and Satisfaction with assistive Technology; Survey S3: System Usability Scale; S4: Supplementary data tables in .CSV format.

## Figures and Tables

**Table 1.** Descriptive statistics of demographic data from the cohort of 30 stroke survivors included in this study. Montreal Cognitive Assessment (MoCA) is an assessment of cognitive ability with a total score of 30 points. Scores of 26–30 indicate normal cognition, 18–25 indicate mild impairment, 10–19 indicate moderate impairment, and 0–9 indicate severe impairment. Days in IRF represent hospital length of stay. The Fugl–Meyer Assessment—Upper Extremity is separated into motor (total = 66 points) and sensory (total = 12 points) parts. See Supplementary Materials for more information.

*N* = 30	Age (Years)	Days in IRF	FMA-UE Motor	FMA-UE Sensory	MoCA
Mean	61.1	13.3	45.6	11.5	25.9
Minimum	25	7	2	5	16
Median	60.5	13	61	12	26.5
Maximum	82	26	66	12	30
Standard Deviation	14.8	5.2	25.1	1.7	4.2

## Data Availability

Survey data presented in this study are included in the Supplementary Materials. Data analysis and visualization code can be accessed at: https://doi.org/10.5281/zenodo.14990338 (4 March 2025).

## Appendix A

Post hoc analyses were performed to compare potential differences in user experience survey results based on cognitive function, as measured by the Montreal Cognitive Assessment (MoCA) score, and motor function, as assessed by the Fugl–Meyer Assessment—Upper Extremity (FMA-UE) score.

For cognition, participants were stratified into two groups: normal cognition (MoCA score ≥ 26; *n* = 19) and cognitive impairment (MoCA score < 26; *n* = 11). Independent *t*-test compared differences in SUS, QUEST, and IMI category (value/usefulness, perceived choice, perceived competence, effort/importance, interest/enjoyment, and pressure). The results did not reach significance in any case (*p* > 0.05), and therefore, we fail to reject the null hypothesis, indicating no significant difference between the groups based on cognition.

For motor function, participants were stratified into two groups: mild impairment (FMA-UE score > 42; *n* = 19) and moderate to severe impairment (FMA-UE score ≤ 42; *n* = 11). An independent *t*-test compared differences in SUS, QUEST, and IMI category scores as described above. Again, the results did not reach significance in any case (*p* > 0.05), and therefore, we fail to reject the null hypothesis, indicating no significant difference between groups based on motor function.

Although our post hoc analyses did not reveal any significant differences in user experience based on cognition or motor function, we acknowledge that our study was not originally designed to adequately compare these groups. Further research with larger sample sizes is warranted to provide more robust evidence that user experience is comparable across cognitive and motor function levels.

## Abbreviations

The following abbreviations are used in this manuscript:

ADLs: Activities of daily living
IRF: Inpatient rehabilitation facility
MI: More involved
LI: Less involved
CIMT: Constraint induced movement therapy
MoCA: Montreal Cognitive Assessment
FMA-UE: Fugl Meyer Assessment—Upper Extremity
SUS: System Usability Scale
QUEST: Quebec User Experience and Satisfaction with Assistive Technology
IMI: Intrinsic Motivation Inventory
