# Supplementary material for: The Feasibility and User Experience of a Program of Progressive Cued Activity to Promote Functional Upper Limb Activity in the Inpatient Rehabilitation Setting with Follow-Up at Home
Source: Appl Sci (Basel). Author manuscript; Available in PMC 2025 Jul 28. (PMC12302696; doi:10.3390/app15063010)
Supplement: Survey S3. System Usability Scale Survey [file NIHMS2092388-supplement-Survey_S3__System_Usability_Scale_Survey.pdf]

This questionnaire will assess the overall usability of the system. Here, the “system” refers to the smartphone app on the provided smartphone and the wearable devices. For each of the 10 items, rate how well you agree with each statement on the following 1-5 scale:

**1**	**2**	**3**	**4**	**5**
strongly disagree				strongly agree

1. I think that I would like to use this system frequently.	1	2	3	4	5
2. I found the system unnecessarily complex.	1	2	3	4	5
3. I thought the system was easy to use.	1	2	3	4	5
4. I think that I would need the support of a technical person to be able to use this system.	1	2	3	4	5
5. I found the various functions in the system were well integrated.	1	2	3	4	5
6. I thought there was too much inconsistency in this system.	1	2	3	4	5
7. I would imagine that most people would learn to use this system very quickly.	1	2	3	4	5
8. I found the system very cumbersome to use.	1	2	3	4	5
9. I felt very confident using the system.	1	2	3	4	5
10. I needed to learn a lot of things before I could get going with this system.	1	2	3	4	5
