# Supplementary material for: The Feasibility and User Experience of a Program of Progressive Cued Activity to Promote Functional Upper Limb Activity in the Inpatient Rehabilitation Setting with Follow-Up at Home
Source: Appl Sci (Basel). Author manuscript; Available in PMC 2025 Jul 28. (PMC12302696; doi:10.3390/app15063010)
Supplement: Survey S1. Intrinsic Motivation Inventory Survey [file NIHMS2092388-supplement-Survey_S1__Intrinsic_Motivation_Inventory_Survey.pdf]

ACTIVITY PERCEPTION QUESTIONNAIRE

The following 37 items concern your experience with the cued exercises from the wearable devices. For each of the following statements, please indicate how true it is for you, using the following 1-7 scale:

1	2	3	4	5	6	7
not at all			somewhat true			very true

Statement	Score (1-7)
1. It was important to me to do well at the cued exercises.	
2. I think that doing the exercises is useful for engaging my arms in exercise.	
3. I tried very hard on the exercises.	
4. I thought the cued exercises were quite enjoyable.	
5. I would describe the cued exercises as very interesting.	
6. I was anxious during the cued exercises.	
7. These were exercises that I couldn't do very well.	
8. I think the exercises are important to do because they can help with my upper-limb rehabilitation.	
9. I am satisfied with my performance at the cued exercises.	
10. I did the cued exercises because I had to.	
11. I did the exercises because I had no choice.	
12. The cued exercises were fun to do.	
13. These were exercises that I couldn't do very well.	
14. After doing the exercises for a while, I felt pretty competent.	
15. I think I am pretty good at performing the exercises.	
16. I believe I had some choice about doing the cued exercises.	

17. I was pretty skilled in doing the cued exercises correctly.	
18. I didn’t try very hard to do well at the cued exercises.	
19. The cued exercises did not hold my attention at all.	
20. I felt very tense while doing the cued exercises.	
21. I enjoyed doing the cued exercises very much.	
22. I think doing this activity could help me to exercise my arms more.	
23. I felt like I had to do the cued exercises.	
24. While I was doing the cued exercises, I was thinking about how much I enjoyed them.	
25. I think I did pretty well with the exercises compared to others who may also be using the same system.	
26. I felt pressure while doing the cued exercises.	
27. I think the exercises are important.	
28. I believe doing the exercises could be beneficial to me.	
29. I did the cued exercises because I wanted to.	
30. I believe the exercises could be of some value to me.	
31. I would be willing to use this exercise system again because it has some value to me.	
32. I didn’t really have a choice about doing the exercises.	
33. I didn’t put much energy into doing the cued exercises.	
34. I put a lot of effort into the cued exercises.	
35. I was very relaxed while doing the cued exercises.	
36. I thought the cued exercises were boring.	
37. I felt like it was not my own choice to do the cued exercises.	

Scoring: Average each subscale. * Items are reverse scored (i.e., subtract reported score from 8).

Interest/enjoyment: 4, 5, 12, 19*, 21, 24, 36*
Effort/importance: 1, 3, 18*, 33*, 34
Value/usefulness: 2, 8, 22, 27, 28, 30, 31
Perceived choice: 10*, 11*, 16, 23*, 29, 32*, 37*
Perceived competence: 7*, 9, 14, 15, 17, 25,
Pressure/tension: 6, 13*, 20, 26, 35*
