# Supplementary material for: The Feasibility and User Experience of a Program of Progressive Cued Activity to Promote Functional Upper Limb Activity in the Inpatient Rehabilitation Setting with Follow-Up at Home
Source: Appl Sci (Basel). Author manuscript; Available in PMC 2025 Jul 28. (PMC12302696; doi:10.3390/app15063010)
Supplement: Survey S2. Quebec User Experience and Satisfaction with Assistive Technology Survey [file NIHMS2092388-supplement-Survey_S2__Quebec_User_Experience_and_Satisfaction_with_Assistive_Technology_Survey.pdf]

The purpose of this questionnaire is to evaluate how satisfied you are with the wearable devices, smartphone app, and provided training you received. For each of the 12 items, rate your satisfaction with the devices or training you experienced with the following 1-5 scale:

**1**	**2**	**3**	**4**	**5**
not satisfied at all	not very satisfied	more or less satisfied	quite satisfied	very satisfied

Provide any comments you have for any of the items under the comments section.

**SMARTPHONE APP AND WEARABLE DEVICES**<br><br>***How satisfied are you with...***	
1. the **dimensions** of the wearable devices (size, height, length, width)?<br>*Comments:*	1 2 3 4 5
2. the **weight** of the wearable devices?<br>*Comments:*	1 2 3 4 5
3. the **ease in adjusting** the wearable devices?<br>*Comments:*	1 2 3 4 5
4. how **safe and secure** the wearable devices are?<br>*Comments:*	1 2 3 4 5
5. the **durability** of the wearable device (endurance, resistance to wear)?<br>*Comments:*	1 2 3 4 5

6. how **easy** is it to use the smartphone app?<br>*Comments:*	1 2 3 4 5
7. how **comfortable** the wearable devices are?<br>*Comments:*	1 2 3 4 5
8. how **effective** the smartphone app and wearable devices are (the degree to which your needs are met)?<br>*Comments:*	1 2 3 4 5

TRAINING AND ASSISTANCE<br><br>***How satisfied are you with...***	
9. the **delivery** (procedures, length of time) of receiving the equipment for this study?<br>*Comments:*	1 2 3 4 5
10. the **troubleshooting** of issues provided by the researchers during setup and use of the app and devices?<br>*Comments:*	1 2 3 4 5
11. the **quality of information** (daily protocol, app instructions) you received for using the app and devices?<br>*Comments:*	1 2 3 4 5
12. the daily **follow-up and communication** from the researchers?<br>*Comments:*	1 2 3 4 5

Below is a list of the same 12 satisfaction items. Please **select THREE items** that you consider to be **most important** to you. Please put an X in **3 boxes** of your choice.

☐ Dimensions

☐ Weight

☐ Adjustments

☐ Safety

☐ Durability

☐ Easy to use

☐ Comfort

☐ Effectiveness

☐ Equipment delivery

☐ Troubleshooting

☐ Instructions

☐ Follow-up communication
